# Cardiac troponins and coronary artery calcium score: a systematic review

**DOI:** 10.1186/s12872-024-03761-x

**Published:** 2024-02-09

**Authors:** Naghmeh Shahraki, Sara Samadi, Omid Arasteh, Reza Javidi Dashtbayaz, Batool Zarei, Amir Hooshang Mohammadpour, Vahid Jomehzadeh

**Affiliations:** 1 Department of Clinical Pharmacy, School of Pharmacy, Mashhad University of Medical Sciences, Mashhad, Iran; 2 Department of Internal Medicine, Faculty of Medicine, Mashhad University of Medical Sciences, Mashhad, Iran; 3 Department of cardiovascular diseases, Faculty of Medicine, Mashhad University of Medical Sciences, Mashhad, Iran; 4 Pharmaceutical Research Center, Pharmaceutical Technology Institute, Mashhad University of Medical Sciences, Mashhad, Iran; 5 Department of Surgery, Faculty of Medicine, Mashhad University of Medical Sciences, Mashhad, Iran

**Keywords:** Troponin T, Troponin I, Cardiac troponins, Coronary calcium score, CAC, Atherosclerosis

## Abstract

An early diagnosis of atherosclerosis, particularly in subclinical status, can play a remarkable role in reducing mortality and morbidity. Because of coronary artery calcification (CAC) nature in radiation exposure, finding biomarkers associated with CAC could be useful in identifying individuals at high risk of CAC score. In this review, we focused on the association of cardiac troponins (hs-cTns) and CAC to achieve insight into the pathophysiology of CAC. In October 2022, we systematically searched Web of Science, Scopus, PubMed, and Embase databases to find human observational studies which have investigated the association of CAC with cardiac troponins. To appraise the included articles, we used the Newcastle Ottawa scale (NOS). Out of 520 records, 10 eligible studies were included. Based on findings from longitudinal studies and cross-sectional analyses, troponin T and I were correlated with occurrence of CAC and its severity. Two of the most important risk factors that affect the correlation between hs-cTns serum levels and CAC were age and gender. The elevation of cardiac troponins may affect the progression of CAC and future cardiovascular diseases. Verifying the association between cardiac troponins and CAC may lead to identify individuals exposed to enhanced risk of cardiovascular disease (CVD) complications and could establish innovative targets for pharmacological therapy.

**Supplementary Information:**

The online version contains supplementary material available at 10.1186/s12872-024-03761-x.

## Introduction

Coronary artery calcium (CAC) is known to be associated closely with atherosclerotic plaque, and predicts the incident of cardiovascular events and mortality [1–3]. It is estimated that by 2035, almost one-half of the population will have cardiovascular diseases, with projected costs of over one trillion dollars [4]. Among the many potentially helpful options, CAC evaluating plays an important role as a risk stratification tool with guideline endorsement for shared decision making in asymptomatic individuals aged 40–75 years, free of atherosclerotic cardiovascular disease (ASCVD) [5, 6]. Moreover, scanning coronary computed tomography (CCT) is capable of reclassifying patients with an intermediate risk for coronary artery disease (CAD), quantifying the specks of calcium within atherosclerotic lesions [7, 8]. By a multi-ethnic cohort of individuals without known CAD with a follow-up of 3.8 years, Detrano et al. demonstrated that the agatson score, reflecting the total area of calcium deposits, is a strong predictor of incident coronary heart disease [9]. Calcium scores under 100 are unlikely to be associated with severe stenosis on coronary angiography and represent a very low risk for obstructive CAD [10, 11]. Nowadays, risk assessment is an important part of routine clinical practice and tools for prediction of CAD events in healthy subjects and the correlated administration of preventive cures have a long history [12]. Cardiac troponins (hs-cTn T and I) are highly sensitive and specific biomarkers which have been shown to be predictive of poorer long-term cardiovascular outcomes in stable patients [13]. These cardiac regulatory proteins control the calcium mediated interaction between actin and myosin. It has been extensively demonstrated that troponin levels play a pivotal role in development of cardiovascular disease, including coronary heart diseases (CHD) [14]. High-sensitivity cardiac troponin (hs-cTn) I and T assays quantify cTn in most healthy men and women and facilitate risk stratification for cardiovascular disease in both acute and outpatient settings [15–17]. With the development of hs-cTn assays, not only CAD but also subclinical CAC can be diagnosed [18]. However, European guidelines still do not recommend general use of cardiac troponins as a risk biomarker [19]. So far, various studies have been conducted to investigate the relationship between hs-cTn serum levels and the CAC diagnosis. Some studies have demonstrated that increased hs-cTn levels in plasma are strongly correlated with CAC risk increasing and CVD [20]. As illustrated in such studies, elevated troponin T levels showed a greater rate of arterial calcification risk [14, 21]. A study that mentioned it in detail demonstrated that an increase above 3 ng/l in hs-cTn T serum level was associated with elevated risk of CAC. However, several investigations failed to indicate the relationship between high hs-cTn T plasma concentration and enhanced risk of CAC. In case of troponin I level, a study was performed on a group of athletes showed that increasing in hs-cTn I plasma level could help to recognize CAC development and further CAD risk stratifications. Moreover, several studies mentioned that an increase in serum troponin I levels was directly related to an increase in agatson score [21, 22]. In this systematic review, we have gathered and overviewed articles that examined the association between serum troponin T and I levels and coronary artery calcium score to see if serum troponins could be considered as reliable factors for diagnosing CAC.

## Methods

### Protocol and registration

The current study followed Preferred Reporting Items for Systematic Reviews and Meta-analyses (PRISMA) statement and was registered in the PROSPERO database (CRD42021246161).

### Search strategy

We searched Pubmed, Web of sciences, Scopus, and Embase with no language and time restrictions to find eligible articles. The keywords used as search bases were obtained from Mesh terms, Emtree terms, and hand searching. The Mesh terms and keywords were obtained from PubMed and Emtree. Our search was conducted with the Mesh terms of cardiovascular diseases, coronary artery disease, coronary disease, troponin, troponin T, and troponin I.

### Inclusion criteria

In the first step, two researchers independently skimmed the articles based on their titles and abstracts. Animal studies, in-vitro experiments, review articles, case reports, clinical trials, editorials, and clinical guidelines were excluded. The conference articles were also excluded due to the lack of required full texts. The studies that did not present a way of comparison were excluded. The only acceptable comorbidities in patients were CVD, type 2 diabetes, metabolic syndrome, and hypertension. As a result, studies that included patients with other comorbidities were excluded to decrease the risk of bias. Furthermore, full texts of the related papers were studied carefully by the same two researchers to see if they were compatible with the inclusion criteria or not. Any disagreements between the two authors were resolved with careful discussion of the third researcher. The inclusion criteria were defined based on the PECO template; population was coronary artery disease and asymptomatic individuals, exposure was cardiac troponins, characterized by elevation of cardiac troponins including troponin I and troponin T, and the outcome was CAC. We defined this template to systematically investigate the observational studies that mentioned the relationship between cardiac troponins and CAC scores.

### Data extraction and quality assessment

Two researchers independently performed data extraction and the following information was extracted from the included studies by the two reviewers: author’s name, year, country of the study population, age, study design, follow-up duration (for cohort studies), study population and number of participants, effect sizes and risk estimates (Odds ratios; OR) with their confidence intervals (CI), and covariates in the multivariable model. Included studies were appraised using Newcastle Ottawa scale (NOS) for observational studies; cohort, case control, and cross-sectional studies. Based on the NOS scale, a score of ≥ 7 is considered good quality. Because of significant heterogeneity among the articles, whether in study design or various cardiac troponins, a meta-analysis on the presented data was failed to conduct.

## Results

### Results of the literature search

**Fig. 1:**
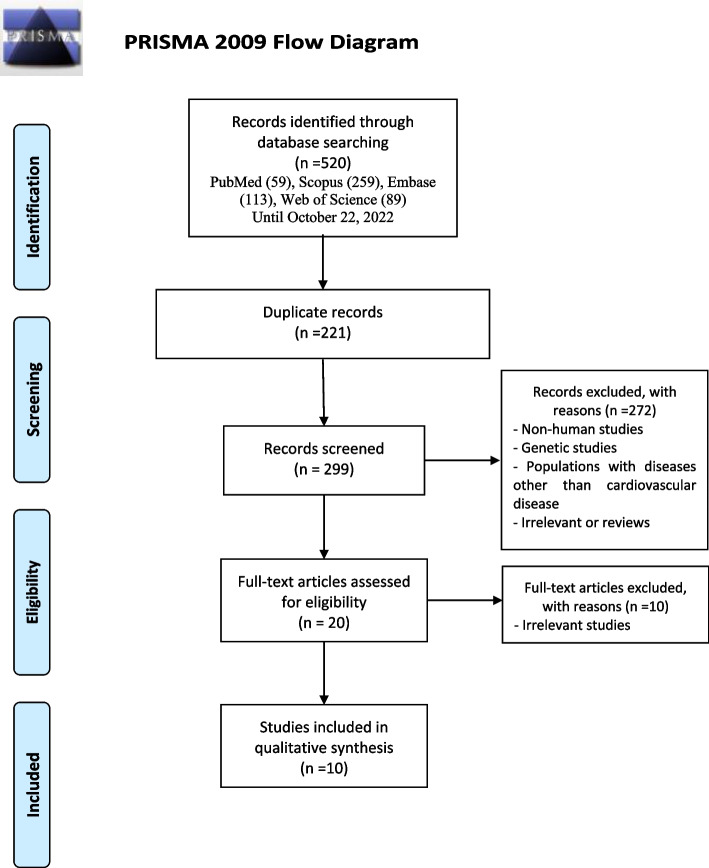
Flowchart of the study

After the screening process, 27 articles seemed potentially eligible based on their titles and abstracts. Three studies used various therapeutic options or electroconvulsive therapy for their patients; therefore, were excluded due to the great risk of bias. Non-English articles were also included except for one Chinese paper, which had a published English duplicate with the same results and more complete data; hence we included the English version. Finally, 10 articles that matched our PECO template were identified for inclusion [17, 18, 21–28]. The complete flowchart of the study selection method is provided as Fig. 1.

### General characteristics of included studies

The majority of the studies in the current review were cross-sectional (*n* = 8) and two were cohort in design [25]. The mean age of participants in the studies ranged from 42 to 76 years. Also, in most of the studies, the male/female ratio in the population of selected participants has been considered, which indicates that the conclusions could be generalized to the community. The effect size of studies varied from 76 patients [21] to 1844 participants [22]. These observational studies were conducted in various continents, but the majority of them were carried out in Europe. So, the results have covered different populations. In all articles, high-sensitivity devices have been used to measure serum hs-cTn levels and serum concentration of hs-cTn I ranged from 1.5 to 32 ng/l and hs-cTn T serum concentration was between 3.46 and 17.9 ng/l. The baseline body mass index (BMI) of the participants indicated that most of the studies examined overweight and obese subjects (BMI > 24 kg/m2). Other studies recruited individuals with mean BMI of less than 30 kg/m2. According to the quality assessments, most of the included studies were classified as good studies.

### Association of cardiac proteins with CAC

The main characteristics of the studies included in the systematic review are summarized in Tables 1 and 2.

**Table 1:** Characteristics of the studies evaluating the association between hs-Tn T and CAC

Author (year)	Country	Age (year)	Population	Study design	Follow-up (year)	Effect size	Finding	Quality assessment
Caselli et al. (2016) [23]	Italy	60.1 ± 0.5	Patients with stable angina and unknown CAD	Cross-sectional, *n* = 297	-	NR	High CAC score was observed in patients with elevated levels of hs-TnT	Good
Rusnak et al. (2017) [21]	Germany	58	Patients with low to intermediate risk of CAD	Cross-sectional, *n* = 76	-	OR(95%CI): 5(1.664–15.025), 0.004^a^ 13.4(1.545–116.233), 0.019^b^ Adjusted for: age, gender, creatinine, uric acid, cholesterol, LDL-C, HDL-C, BMI, triglycerides, arterial hypertension, cardiac family history, smoking, diabetes and NT-proB	Agatston score was significantly correlated with hs-TnT, both in univariable and multivariable linear regression models	Moderate
Kitagawa et al. (2015) [24]	Japan	69.2 (9.8)	Stable patients with clinical suspicion of CAD	Cross-sectional, *n* = 215		OR(95%CI): 1.250 (1.15–1.378), < .0001^c^ 1.101(1.054–1.157)^b^ Adjusted for: age, sex, BMI, average systolic blood pressure, hemoglobin A1c, total cholesterol, logarithm of triglycerides, uric acid, creatinine, smoking status	Serum hs-cTnT is associated with coronary calcium in individuals with suspected coronary disease	Moderate
Razavi et al. (2021) [25]	USA	58.9	Participants with MetS or T2DM	Cohort, *n* = 574	10	OR(95%CI): Total: 1.55(1.01–2.38),0.04 MS: 1.37(0.84, 2.24),0.2 T2DM: 3.35(1.22, 9.15),0.02 Adjusted for: age, sex, race, education, antihypertensive medication, lipid-lowering medication, glucose-lowering medication, cigarette smoking, waist circumference, blood pressure, fasting blood glucose, fasting serum triglycerides, and total cholesterol/HDL-C ratio	Individuals with a serum hs-cTnT concentration < 3 mg/dL had 55% higher odds of long-term absence of CAC compared to those with a hs-cTnT concentration ≥ 3 mg/dL (*p*-value = 0.04)	Good
Tveit et al. (2022) [26]	Norway	65	Patients referred for angiographic evaluation of CAD	Cross-sectional, *n* = 646	-	B: 0.52(0.25–0.79), *p* < 0.001 Adjusted for: age, sex, current smoking, history of CAD, diabetes or HF, BMI, SBP, LDL-C and eGFR	There was a graded linear association between higher concentrations of hs-cTnT and higher CAC-score in the total population.	Good
Lazzarino et al. (2015) [27]	UK	62.8(5.7)	Disease-free, low-risk participants	Cross-sectional, *n* = 430	-	NR	the more accurate a score is in predicting detectable HS-CTnT, the less it is mediated by CAC	Moderate
Sandoval et al. (2020) [17]	US	62(10)	Free of clinical CVD	Cohort, *n* = 6749	15	Cohen’s k: 0.24(0.22–0.26)	Concordance between undetectable/detectable hs-cTnT and CAC demonstrated an agreement rate of 62%, which varied slightly by race/ethnicity.	Good
Cardinaels et al. (2016) [18]	Netherlands	55.8 ± 11.0	Patients with chest discomfort	Cross-sectional, *n* = 1864	-	NR	hs-cTnT concentrations were associated with coronary calcium score.	Good

*OR* odds ratio, *T2DM* type 2 diabetes, *CAC* coronary artery calcification, *CVD* cardiovascular disease, *CCS* coronary calcium score, *CAD* coronary artery disease, *NR* not reported, *hsTnI* high-sensitivity troponin T, *MS* metabolic syndrome, *HF* heart failure, *BMI* body mass index, *SBP* systolic blood pressure, *HDL-C* high density lipoprotein cholesterol, *LDL-C* low density lipoprotein cholesterol, *eGFR* estimated glomerular filtration rate

^a^Agatston score > 100

^b^Agatston score > 400

^c^Agatston score > 10

**Table 2:** Characteristics of the studies evaluating the association between hs-Tn I and CAC

Author (year)	Country	Age (year)	Population	Study design	Follow-up	Effect size	Finding	Quality assessment
Januzzi et al. (2019) [22]	USA	59.7(7.8)	Symptomatic outpatients with suspected CAD	Cross-sectional *n* = 1844	-	NR	Stable outpatients with suspected CAD, concentrations of hsTnI were significantly associated with CAC as well as obstructive CAD.	Good
Rusnak et al. (2017) [21]	Germany	58	Patients with low to intermediate risk of CAD	Cross-sectional *n* = 76	-	OR(95%CI): 3.4(0.867–13.337), 0.07^a^ 8.8(1.183–65.475), 0.034^b^ Adjusted for: age, gender, creatinine, uric acid, cholesterol, LDLC, HDLC, BMI, triglycerides, arterial hypertension, cardiac family history, smoking, diabetes and NT-proB	hs-cTnI was increasing alongside Agatston score and was able to differentiate between different groups of Agatston scores.	Moderate
Tveit et al. (2022) [26]	Norway	65	Patients referred for angiographic evaluation of CAD	Cross-sectional *n* = 646	-	B: 0.68(0.43–0.93), *p* < 0.001 Adjusted for: age, sex, current smoking, history of CAD, diabetes or HF, BMI, SBP, LDL-C and eGFR	There was a graded association between higher concentrations of hs-cTnI and higher CAC-score in the total population. This association was non-linear for hs-cTnI with a stronger association below 3 ng/L.	Moderate
Olson et al. (2016) [28]	Denmark	49% were 50 years old	Middle-aged subjects without known CVD	Cross-sectional *n* = 1173	-	OR(95%CI): 1.25(1.03–1.51), 0.025^c^ 1.36(1.08–1.71), *p* = 0.009^a^ 1.22(0.88–1.69), 0.2^b^ Adjusted for: sex, age, hypertension, hypercholesterolemia, smoking, diabetes, family history of CVD and creatinine	Hs-TnI was associated with CAC in a Danish middle-aged population without previously known CVD. This is a step towards understanding hs-TnI as a risk marker for CVD.	Good
Cardinaels et al. (2016) [18]	Netherlands	55.8 ± 11.0	Patients with chest discomfort	Cross-sectional *n* = 1864	-	NR	hs-cTnI concentrations were associated with coronary calcium score.	Good

*OR* odds ratio, *T2DM* type 2 diabetes, *CAC* coronary artery calcification, *CVD* cardiovascular disease, *CCS* coronary calcium score; *CAD* coronary artery disease, *NR* not reported, *hsTnI* high-sensitivity troponin T, *MS* metabolic syndrome, *HF* heart failure, *BMI* body mass index, *SBP* systolic blood pressure, *HDL-C* high density lipoprotein cholesterol, *LDL-C* low density lipoprotein cholesterol, *eGFR* estimated glomerular filtration rate

^a^Agatston score > 100

^b^Agatston score > 400

^c^Agatston score > 0

#### Troponin T and CAC association

All of the studies that have examined the relationship between hs-cTn T and CAC score concluded that the more serum hs-cTn T concentration increased, the more amount of agatson score rose. In one cross-sectional study that assess the relationship between hs-cTn T and CAC score, 229 male patients with stable angina and unknown CAD were studied. In these 60 years old patients, higher CAC scores were seen in patients with significantly elevated levels of hs-Tn T (*P* < 0.005). Also, in a multivariate model, CAC score was an independent predictor of the plasma hs-cTn T (coefficient = 0.06, SE = 0.02; *P* = 0.0089). Overall, this study concluded that the presence and extent of coronary atherosclerosis is associated noticeably with the circulating levels of hs-cTn T [23]. In another cross-sectional study, 215 consecutive, stable patients with clinical suspicion of coronary artery disease were enrolled. It is demonstrated a clear significant association between serum hs-cTn T (LoD: 3 ng/L) concentrations and subclinical atherosclerosis degree as determined by coronary calcium and expressed through the agatson score. One of the limitations of this study was that the participants enrolled were 69 years old Japanese men and women. Therefore, these findings may not be generalized to other ethnic groups. Also, more participants are needed to warrant the study results [24]. In a study by Alexander C. Razavi et al., 574 patients with D2TM (*n* = 152) or metabolic syndrome (*n* = 422) at baseline were selected from the MESA cohort and their CAC levels were prospectively evaluated. Two third of the study population were women and the average age was 58.9 years old. It was clear that the participants who had the long-term absence of CAC were younger and they had lower fasting blood glucose and hs-cTn T level. In addition, those with the CAC score of zero did not have a carotid artery plaque. Also, 55% higher odds of long-term absence of CAC was observed in patients with serum hs-cTn T concentration < 3 mg/dl as compared with those with hs-cTn T ≥ 3 mg/dl (*p* = 0.04). The results of this study showed that hs-cTn T level elevation may reflect both subclinical myocardial injury and systemic arterial stiffness in persons with metabolic disease. As a result of this study, an increase in hs-cTn T levels and severity of metabolic syndrome was considered as potential ASCVD risk factors which could predict the arterial aging and CAC [25].

Lazzarino et al. recruited 430 participants drawn from the Whitehall II epidemiological cohort and aged 53–76 years with no history of clinical or subclinical CVD and no previous diagnosis or treatment for hypertension, inflammatory diseases, allergies, or kidney disease to evaluate the effectiveness of the Framingham, Joint British Societies & British National Formulary (JBS/BNF), Assign, and Q-Risk 2 scores in identifying subjects with detectable hs-CTn T in circulation. They also determined whether the scores’ estimates are influenced by CAC and to what extent. Their founding illustrated that if the mentioned risk algorithms are arranged based on the ROC areas, the age and gender model has the highest ranking, followed by Q-Risk2, Framingham, JBS/BNF, and Assign. Nevertheless, when the scores are arranged regarding the degree of mediation by CAC, an essentially reversed order could be seen. This implies that as the accuracy of a score in predicting detectable hs-CTn T increases, its dependence on CAC as a mediator decreases. Alternatively, a score that effectively identifies atherosclerosis has a reduced ability to predict cardiac damage (*P* = 0.009) [27]. Study by Sandoval et al. has examined the relationship between hs-cTn T and CAC severity in 6,749 participants free of clinical cardiovascular disease at baseline during 15 years. In this study, it was identified that participants with detectable CAC had a higher incidence rate of CVD than those with undetectable CAC. Also, individuals with traceable hs-cTn T (> 3 ng/l) had a higher CAC level. Moreover, it was shown that hs-cTn T was an independent risk factor for CVD incidence in multivariable Cox regression analyses. In the adjusted analysis models, it was found that the relationship between detectable hs-cTn T and CVD is significant mostly in women not in men (HR: 1.7 vs. 1.49) [17]. These results extend the value of hs-cTn T, which is a prognostic factor for short and long term CVD outcomes.3.3.2. Studies measured both troponins (T and I) and CAC.

A cross-sectional study on 76 consecutive patients undergoing CCT during routine clinical care was done prospectively to measure the cardiac biomarkers, hs-cTn T and hs-cTn I concentrations (LoD: 0.005 µg/l and LoD: 1.1–1.9 ng/l respectively) in association with CAC. In other words, in both univariate and multivariate logistic regression models, hs-cTn biomarkers were significantly correlated with increased agatson scores. One of the limitations of this study was the small sample of patients that could not be proposed for the general population. Moreover, the people who were selected from the PROMISE trial were mostly Caucasian individuals that did not reflect multiethnic cohorts [21]. In another study, 706 patients with 65 years old age who suspected chronic coronary syndrome (CCS) and were undergone for angiographic evaluation of CAD checkup were examined. It was depicted that both hs-cTn concentrations were significantly higher in CAD50 patients than in non-obstructive CAD and the ones without CAD (*p* < 0.001). Although the higher concentrations of hs-cTn I and T were related to CAD50 in unadjusted analysis (OR 1.45, 95% CI [1.28–1.64], *p* < 0.001, hs-cTn T: OR 1.27 [1.13–1.41], *p* < 0.001), it was mentioned that just hs-cTn I concentration was significantly associated with CAD50 after adjustment for age, sex, smoking, history of CAD, diabetes and HF, BMI, SBP, LDL-C and eGFR (OR 1.20 [1.05–1.38], *p* = 0.009) [26]. Another cross sectional study on 1864 individuals with chest pain discomfort was performed by Cardinaels et al. which evaluated the hs-cTn T and hs-cTn I concentrations in relation with CAC. The average age of these patients was about 55.8 ± 11.0 and the ratio of men to women was 56.0%. It was shown that hs-cTn concentrations were remarkably associated with the coronary calcium score according to both univariate and multivariate linear regression analysis (*P* < 0.001) [18].

#### Troponin I and CAC association

A study by James L. Januzzi et al. conducted on 1844 stable symptomatic outpatient and revealed that hs-Tn I level was associated with the transition from non-calcified to calcified vascular plaque. The authors adjusted correlations for differences in age, and gender. It is suggested that higher circulating hs-Tn I levels were more related to the CAD progression prospectively with no dependence on other patient characteristics. Moreover, higher hsTn I concentrations were a predictive factor for moderate and severe coronary obstruction. CAC scores exhibited weak bivariate correlation with log hsTn I when added to multivariable linear regression models [22]. Moreover, the relationship between hs-Tn I and CAC was evaluated by Olson et al. using logistic regression analyses and receiver operating characteristic curves (ROC). This investigation was performed on 1173 randomized, middle-aged subjects without known CVD, indicating 29% presence of CAC (agatson score > 0) in the lowest quartile of hs-Tn I compared to 55% at the highest rate, with a step-by-step increase over quarters. The Spearman correlation coefficient between hs-Tn I and CAC was 0.23, which showed the strong correlation between these two factors (*p* < 0.0001) [28].

#### Troponins (T and I) and CAC risk in asymptomatic individuals

In a population-based cross-sectional study with normally gender distributed patients aged 58 years old or above, participants undergoing coronary computed tomography (CCT) as part of their routine clinical care were consecutively included. According to the results, in these cardiovascular asymptomatic patients, the more CAC was measured the more concentration of both hs-Tn T and I was reported both in univariable and multivariable linear regression models. Individuals who had high levels of hs-cTnT (≥ 0.02 µg/l) and hs-cTnI (≥ 5.5 ng/l) were more prone to displaying CAC values ≥ 400 [21].

Another study by Lazzarino et al., recruiting disease-free subjects suggests that as the accuracy of a score in predicting detectable hs-CTnT increases, its reliance on CAC as a mediator decreases. In other words, a score that effectively identifies atherosclerosis has a diminished ability to predict cardiac damage. A limitation of this study is it’s cross-sectional nature, in which the evaluation of hs-cTn T was not considered in a prospective manner and it was not exempt from selection bias [27]. In a prospective cohort study of Multi-Ethnic Study of Atherosclerosis (MESA) with median follow-up of 15 years, 1,002 ASCVD incidents occurred among 6,749 individuals free of clinical CVD with a mean age of 62 (10) years and 53% women. It was shown that subjects with detectable hs-cTnT (HR, 1.47; 95% CI, 1.21–1.77; p 0.001) and detectable CAC (HR, 2.35; 95% confidence interval [CI], 2.0 -2.76; p 0.001) possessed increased rates of ASCVD compared with undetectable findings. Similarly, participants with undetectable hs-cTnT (32%) and subjects with zero CAC (50%) both showed comparable risks for ASCVD. Therefore, utilizing both markers together enhances the accuracy of risk prediction [17]. Additionally, in a cross-sectional study, 1173 asymptomatic participants were chosen at random from the Danish community; 52% of them were female and between the ages of 50 and 60. Logistic regression analyses were used to determine the distribution of the agatson score and hs-TnI quartiles throughout the entire population. Results showed that the differences in hs-TnI and CAC between men and women were statistically significant (*p* < 0.0001). When employing hs-TnI quartiles as a predictor, univariate regression revealed that for all dichotomous CAC outcomes, being in a higher hs-TnI quartile carried a stepwise increased chance of having a greater CAC burden. When adjusting for cardiovascular risk factors, being in the highest hs-TnI quartile led to a 56% increased risk of having an agatson score > 0 and a 82% enhanced risk of having an agatson score > 100 when compared to the lowest quartile. However, Hs-TnI was not able to predict an agatson score > 400. An increase of 1 in the log-transformed hs-TnI led to a 27% accelerated risk for falling into a higher CAC category after adjustment for risk factors [28].

## Discussion

As CAC measurement is rather expensive and implies radiation exposure, this study aims to describe clinical evidence in case of examining the prognostic role of cardiac troponins in determining the risk of CAC. We systematically reviewed ten cross-sectional (*n* = 6545) and two cohort (*n* = 7323) studies regarding association of cardiac troponins and CAC. Variability of the results between included studies might be the result of difference in methodological design and patient characteristics. Despite variables such as population sample sizes, age, inclusion criteria, primary inflammatory markers studied, and analysis, several studies reported a significant correlation between level of plasma troponin and CAC existence or severity.

The pathophysiological mechanism behind artery calcification has remained unresolved and so the role of various biomarkers such as troponin plasma levels in the process is yet difficult to identify. Coronary artery calcification may occur in different situations and the involved signaling pathways are variously changed in different clinical status. Generally, there are several mechanisms proposed to explain vascular calcification including induction of bone formation, circulating nucleational complexes, and cell death [29]. Analyses from Cox regression models in a large cohort study by Sandoval et al. with 15-year follow-up and 6,749 participants without cardiovascular disease at baseline has identified that individuals with higher levels of hs-cTn T were subjected to 15.4 events of CVD incidence against 5.2 events for lower hs-cTn T concentrations per 1,000 person-years [17]. These results highlight the value of detectable/undetectable CAC/hs-cTn T evaluation as a robust prognostic factor for short and long term ASCVD outcomes (20% vs. < 3%). The most important advantages of this study were the long length of observation and evaluation in the multi-ethnic community. In a prospective study by Razavi et al., the healthy arterial aging in individuals with a background of metabolic syndrome or diabetes mellitus was evaluated for 10-years follow-up and the rate of CAC score changes was measured. It was concluded that although the absence of cardiovascular risk factors does not play a role in the rate of CAC progression, the level of hs-cTn T could be a good factor in predicting artery calcification [25].

Furthermore, there are several cross-sectional studies that showed the association of hsTn T with the incidence and the progression of CAC score was significantly remarkable [18, 21, 24]. On the other hand, a study by Paana et al. showed a lack of correlation between hsTn T and incidence of CAC. However, the small number of participants and their selection from the marathon runners did not represent the whole community in this study which was an important limitation factor [30]. In order to examine the relationship between hsTn I and CAC severity, Cardinaels et al. represented that hs-cTn I concentrations are significantly correlated with the incidence of CAC [18]. Also, a study that was done by Januzzi et al. on 1844 stable symptomatic outpatients without known CAD concluded that in case of high concentrations of hsTn I, more prevalent and more extensive obstructive CAD was observed with higher CAC scores [22]. To better understand this relationship, the study should be performed on a more diverse and larger population.

Some potential reasons for discrepancies in articles’ results were explained by study design and methodological issues, variability in population characteristics and ethnicity, sample size, gender, and method of measuring. Although most studies have used high sensitive methods to measure serum troponin levels, it is difficult to assess low serum troponin concentrations in asymptomatic individuals, and this may be a reason for differences in results. According to the results of the studies, two of the most important risk factors that affect the correlation between hs-cTns serum levels and CAC were age and gender. As Lazzarino et al. mentioned, CAC mediated 6.8% of the impact of age and gender on hs-CTn T in participants without CAC at baseline [27]. Moreover, in Rusnak et al. study, it has been shown that in different agatson categories, the average age as well as the levels of uric acid are increasing according to rising agatson values which indicated CAC is in the relation with age [21]. The effect of age is also mentioned in the Kitagawa et al. study in relation with hs-cTn T serum level and both agatson score > 10 and > 400. However, multiple regression analyses demonstrated that serum hs-cTn T increased the odds of both agatson score > 10 and 400 [24]. Additionally, Cardinaels et al. stated that age is considered as an independent predictor for 30% and 19% of hs-cTn T and hs-cTn I variation respectively [18]. It is specified in this article that only age, smoking and total cholesterol were significantly different in the event versus non-event group. This is in accordance with the study by Paana et al., which mentioned the significant correlation of age and hs-cTn T concentrations after a run race among athletes [30]. Ethnicity, also, may play a role in the conflicting results of the studies in this review. It is identified that a weak association between hs-cTn I and CAC was found among studies in which the majority were white population [30]. Although measurements of serum hs-cTn levels have immense promise as predictive markers for future CHD [31], currently, there is a lack of strong evidence that they add significantly to global risk assessment. To achieve more precise results, high-qualified prospective studies with matched designs are required to minimize the risk of bias. Nevertheless, this study has some limitations. The included studies were observational, which increases the possibility of bias. Moreover, the design of the studies had some differences which can affect the results. Further high-quality longitudinal studies with larger populations are required to prove these findings. In addition, for future clinical studies, researchers should consider the presence of confounding variables and adjust their study designs to get more accurate results.

## Conclusions

The increase of cardiac troponins level may enhance the risk of coronary calcification and future cardiovascular outcomes. Verifying the association between cardiac troponins and CAC may assist to identify individuals susceptible to enhanced risk of CVD complications and could establish innovative targets for pharmacological therapy.

### Supplementary Information

**Additional file 1.**

## Data Availability

The datasets used and/or analysed during the current study available from the corresponding author on reasonable request.
